# Analysis of seed production and seed shattering in a new artificial grassland forage: pigeon pea

**DOI:** 10.3389/fpls.2023.1146398

**Published:** 2023-05-12

**Authors:** Xinyong Li, Wei Sheng, Qianzhen Dong, Rui Huang, Rongshu Dong, Guodao Liu, Xipeng Ding, Jingwen Zhang

**Affiliations:** ^1^ Institute of Tropical Crop Genetic Resources, Chinese Academy of Tropical Agricultural Sciences, Danzhou, China; ^2^ College of Tropical Crops, Hainan University, Haikou, China; ^3^ Ministry of Education Key Laboratory for Ecology of Tropical Islands, College of Life Science, Hainan Normal University, Haikou, China

**Keywords:** pigeon pea, seed yield, yield component, structural equation model, seed shattering, abscission layer

## Abstract

Pigeon pea is a perennial leguminous plant that is widely cultivated as a forage and pharmaceutical plant in subtropical and tropical areas, especially in artificial grasslands. Higher seed shattering is one of the most important factors in potentially increasing the seed yield of pigeon pea. Advance technology is necessary to increase the seed yield of pigeon pea. Through 2 consecutive years of field observations, we found that fertile tiller number was the key component of the seed yield of pigeon pea due to the direct effect of fertile tiller number per plant (0.364) on pigeon pea seed yield was the highest. Multiplex morphology, histology, and cytological and hydrolytic enzyme activity analysis showed that shatter-susceptible and shatter-resistant pigeon peas possessed an abscission layer at the same time (10 DAF); however, abscission layer cells dissolved earlier in shattering-susceptible pigeon pea (15 DAF), which led to the tearing of the abscission layer. The number of vascular bundle cells and vascular bundle area were the most significant negative factors (*p<* 0.01) affecting seed shattering. Cellulase and polygalacturonase were involved in the dehiscence process. In addition, we inferred that larger vascular bundle tissues and cells in the ventral suture of seed pods could effectively resist the dehiscence pressure of the abscission layer. This study provides foundation for further molecular studies to increase the seed yield of pigeon pea.

## Introduction

Pigeon pea (*Cajanus cajan* (Linn.) Millsp.) is a perennial leguminous plant with a wide range of uses because it tolerates drought and barren soil. It can be used as a forage and pharmaceutical plant (Khuntia et al., 2022), and pigeon pea is widely planted in subtropical and tropical regions and plays a major role in artificial grasslands. However, higher seed shattering is one of the most important factors in potentially increasing the seed yield of pigeon pea.

Seed production is very important in all forage species. The seed yield of perennial forage is affected by the contribution of its components (Bhatt, 1973). Screening key yield components is conducive to improving seed yield (Nouraein, 2019). Using path analysis and regression analysis, we can determine a one-way causal relationship between the yield components of sequential development, that is, the causal relationship between yield components and seed yield. This relationship has been determined in many crops, such as soybean (*Glycine max* (L.) Merrill.), perennial ryegrass (*Lolium perenne* L.), red clover (*Trifolium pratense* L.), and chickpea (*Cicer arietinum* L.) (Jain et al., 2015; Abel et al., 2017; Amdahl et al., 2017; Chopdar et al., 2017).

Pod shattering refers to the phenomenon in which the ventral or dorsal suture of the pod splits at the mature stage, causing seeds to spread (Parker et al., 2020). It is often found in Cruciferae (Rodriguez-Gacio et al., 2004; Qing et al., 2021) and Leguminosae (Christiansen et al., 2002; Ma et al., 2020). Pod shattering is a major means of reproduction for plants themselves, but this atavistic phenomenon is not conducive to seed production (Abd El-Moneim, 1993). There is a certain relationship between the morphological characteristics of pods shatter resistance (Braatz et al., 2018). The mechanical force ability of the abscission layer to withstand external factors is weaker when the dehiscence degree is stronger (Jia et al., 2021). Importantly, the abscission layer is located at the junction of the pod ventral and dorsal sutures. With pod growth, the water content of the pod tends to rise first and then decline (Kuai et al., 2016). The pectin of the pod abscission layer is decomposed by cellulase (CE) and polygalacturonase (PG), which together promote pod dehiscence (Dong et al., 2019; Guo et al., 2022). Meanwhile, the key cell structures (fiber cap cells, outer valve marginal cells) controlling pod dehiscence have provided new insight (Dong et al., 2014; Dong et al., 2017b). These results show that pod dehiscence is under the joint regulation of factors such as the apparent morphological characteristics, plant cell structure, physical conditions, physiology and biochemistry, and external environment.

In this study, we observed 21 morphological characteristics of 70 pigeon pea accessions to explore the relationship between yield components and seed yield and the effect of morphological characteristics on pigeon pea pod dehiscence. Among accessions, by comparing differences in pod dehiscence, multiplex morphology, and physiological and anatomical structure of pods, the mechanism of pod dehiscence in pigeon pea was systematically and comprehensively studied.

## Materials and methods

### Plant material

The 70 pigeon pea accessions used in this study (**Table 1**) were grown at the forage base of the Chinese Academy of Tropical Agriculture Sciences, Hainan, China (N 19°30′, E 109°30′, 149 m above sea level). The mean annual precipitation is 2229 mm.

**Table 1:** Basic information on the 70 pigeon pea accessions.

Code	Accessions	Source of seed	Code	Accessions	Source of seed
1	D21001	Hainan	36	D21036	Guangdong
2	D21002	Hainan	37	D21037	Hainan
3	D21003	Hainan	38	D21038	Yunnan
4	D21004	Hainan	39	D21039	Hainan
5	D21005	Hainan	40	D21041	India
6	D21006	Hainan	41	D21042	Hainan
7	D21007	Hainan	42	D21043	Hainan
8	D21008	Hainan	43	D21045	India
9	D21009	Guangxi	44	D21046	Guangdong
10	D21010	Hainan	45	D21047	Hainan
11	D21011	Hainan	46	D21048	Hainan
12	D21012	Hainan	47	D21049	Guangxi
13	D21013	Guangxi	48	D21050	Yunnan
14	D21014	Jiangxi	49	D21051	Guangdong
15	D21015	Jiangxi	50	D21054	Guangxi
16	D21016	Guangxi	51	D21055	India
17	D21017	Guangxi	52	D21056	Hainan
18	D21018	India	53	D21057	Yunnan
19	D21019	India	54	D21059	Yunnan
20	D21020	India	55	D21061	Hainan
21	D21021	Hainan	56	D21063	Yunnan
22	D21022	Fujian	57	D21065	Hainan
23	D21023	Hainan	58	D21066	Yunnan
24	D21024	Hainan	59	D21067	Yunnan
25	D21025	Hainan	60	D21076	Yunnan
26	D21026	Hainan	61	D21080	Yunnan
27	D21027	Hainan	62	D21081	Hainan
28	D21028	Guangdong	63	D21084	Guangdong
29	D21029	Hainan	64	D21086	Yunnan
30	D21030	Hainan	65	D21090	Yunnan
31	D21031	Hainan	66	D21094	Myanmar
32	D21032	Hainan	67	D21095	Myanmar
33	D21033	EL Salvador	68	D21099	EL Salvador
34	D21034	Hainan	69	D21100	Pakistan
35	D21035	Hainan	70	D21103	Kenya

### Experimental design and seed yield components

The experiment began in April 2021. One experimental plot was planted for each germplasm material, and 12 individual plants were established in each plot. After 2 years of field observation, 10 shatter-susceptible accessions were selected, and the dehiscence characteristics were stable. Statistical analysis of field agronomic characteristics was carried out in 2021. After reaching the full flowering stage, 5 pigeon peas were randomly selected from each experimental plot for marking. Yield components were fertile tiller number per plant (FTP), inflorescences per tiller (IT), flowers per inflorescence (FI), ovules per flower (OF) and thousand-seed weight (TSW). The actual seed yield per plant was calculated for 5 plants taken from the center of each plot. Seeds were air-dried to 6 to 8% moisture before seed yield was calculated. The potential seed yield (PSY) per plant was determined by the following equation:


PSY = (FTP×IT×FI×OF×TSW)/1000

### Morphological characteristics

Plant height, crown breadth, number of primary branches, number of secondary branches, fertile tillers, length and width of the middle leaflets on triple leaves, flowers per inflorescence, inflorescences per tiller, and ovules per flower were measured at the flowering stage of the plant. In the seed maturation stage, pod width, length, and thickness, the number of seeds per pod, and plant biomass were measured.

### Characteristics of pod dehiscence

The mechanical force of pod dehiscence was measured by an Adelberg HP-50 digital display push-pull meter, including vertical and horizontal pod mechanical forces. Fifty mature pods of each accession were dried for several weeks until the pods naturally dehisced (without manual intervention), and the pod shattering rate was determined. The torsion laps of the pod were measured by taking the pod wall as a circle with 360 degrees of upward spiral.

### Features of the abdominal suture

Two shatter-susceptible accessions (D21001, D21034) and two shatter-resistant accessions (D21023, D21025) were selected. The samples were obtained at 10, 15, 20, 25, 30, and 35 days after flowering (DAF) and were saved in FAA (formaldehyde alcohol acetic acid) fixative for paraffin sectioning of pod ventral sutures. The plant tissues were stained for 25 min with 0.7% toluidine blue. Then, the target area was selected with CaseViewer 2.4 scanning software for 120 images. After imaging, Image-Pro Plus 6.0 software was used to measure the single vascular bundle cell area, abscission layer cell area, vascular bundle area, width of the vascular bundle area and single vascular bundle cells, length of the vascular bundle area and number of vascular bundle cells.

### Physiological characteristics

The pod wall and seed of each pod were separated at 20, 25, 30, and 35 DAF to calculate water content. Enzyme activity was determined with a cellulase and polygalacturonase activity detection kit (Beijing Solarbio Science & Technology Co., Ltd.).

### Data and statistical analysis

Microsoft Excel was used for data entry and tables. Data analysis was done with DPS 7.05 (DPS Inc., USA) software and origin2022bsr1 was used for figures, A 3DHISTECH (Hungary) panoramic slice scanner was used for tissue sectioning, 3DHAISTECH (Hungary) CaseViewer 2.4 was used for tissue observation, and Media Cybemetrics (U.S.A.) Image-Pro Plus 6.0 was used for tissue data analysis. Structural equation modeling (SEM) is a powerful tool for examining relationships between causally linked intercorrelated variables. Each single-headed arrow in a structural equation model represents a causal relationship where the variable at the tail of the arrow is a direct cause of the variable at the head.

## Results

### Yield components and seed yield

From the yield components and seed yield (**Table 2**), we found that the average actual seed yield was only 6% of the potential seed yield, indicating that there is great potential to increase seed yield in pigeon pea. The small coefficient of variation for flowers per inflorescence indicated that this component had better overall stability. The variation coefficients for inflorescences per tiller and fertile tiller number per plant were relatively large, indicating that the seed yield is largely affected by these two components.

**Table 2:** Analysis of seed yield and yield components.

Index	FTP	IT	FI	OF	TSW (g)	SY (g)	PSY (g)	SY/PSY (%)
Maximum	152.7	26.3	8.3	5.8	177.8	349.5	7875.8	14
Minimum	21.0	5.5	7.3	3.9	77.5	31.1	793.8	1
Average value	79.0	11.6	7.9	4.8	99.2	156.8	3219.3	6
Standard deviation	28.3	5.4	0.3	0.4	16.2	68.9	1615.6	3
Variable coefficient (%)	35.9	46.2	4.3	7.5	16.3	43.9	50.2	50.5

**Figure 1:**
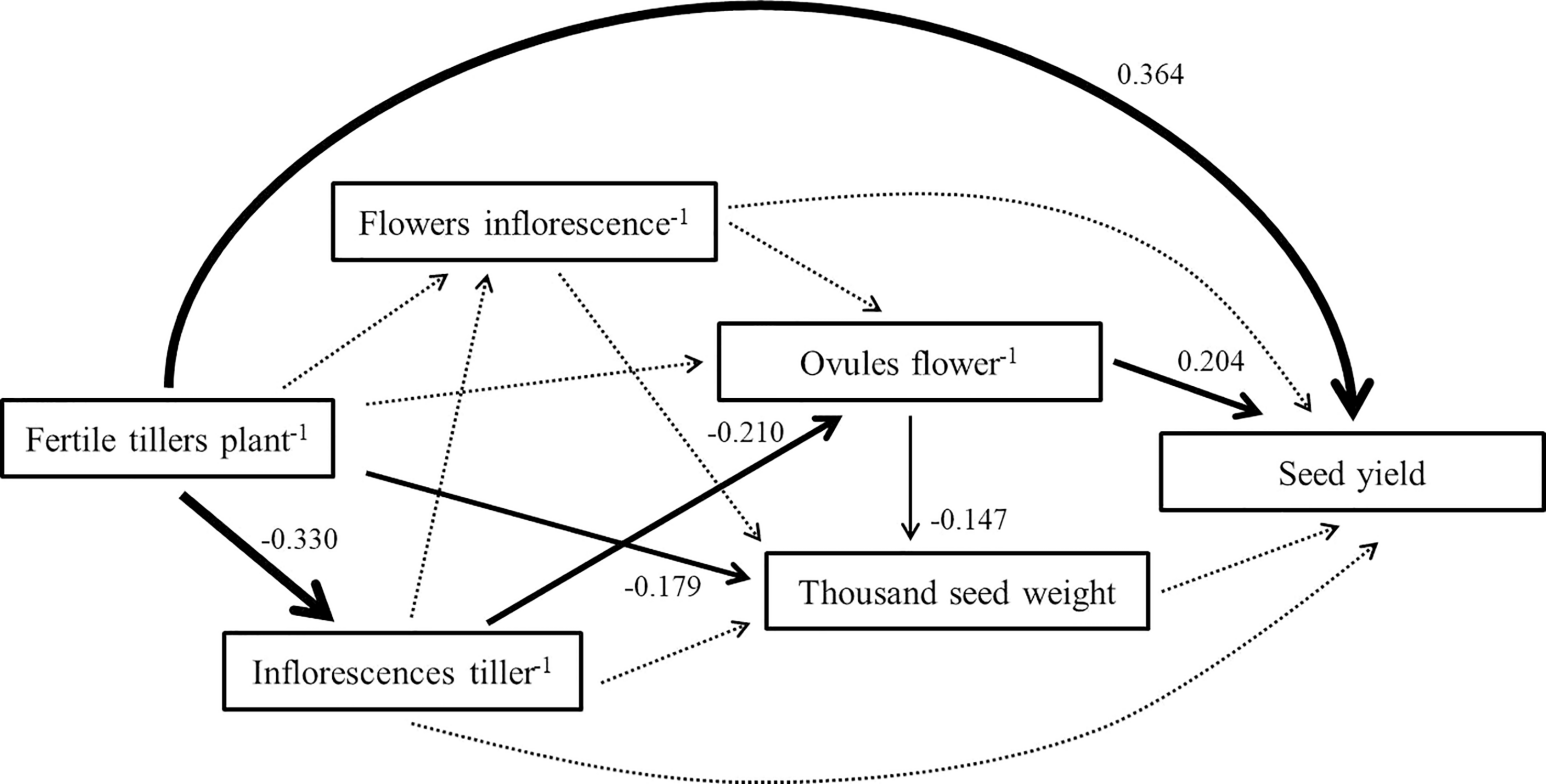
The structural equation model linking pigeon pea seed yield to yield components. Each arrow illustrates a relationship in which the change in one variable at its head directly causes the change in another variable at its tail. Dotted arrows represent nonsignificant pathways. Greater standardized coefficients (given beside each significant path) show a stronger relationship between the variable at the head and the variable at the tail.

The SEM showed that the direct effect of fertile tiller number per plant (0.364) on pigeon pea seed yield was the highest of the 5 components (**Figure 1**). Furthermore, the negative indirect effect of inflorescences per tiller (-0.330), based on fertile tiller number per plant, was large. No indirect or direct effect was observed for flower number per inflorescence or thousand seed weight.

### Mechanical characteristics of the pod

**Figure 2:**
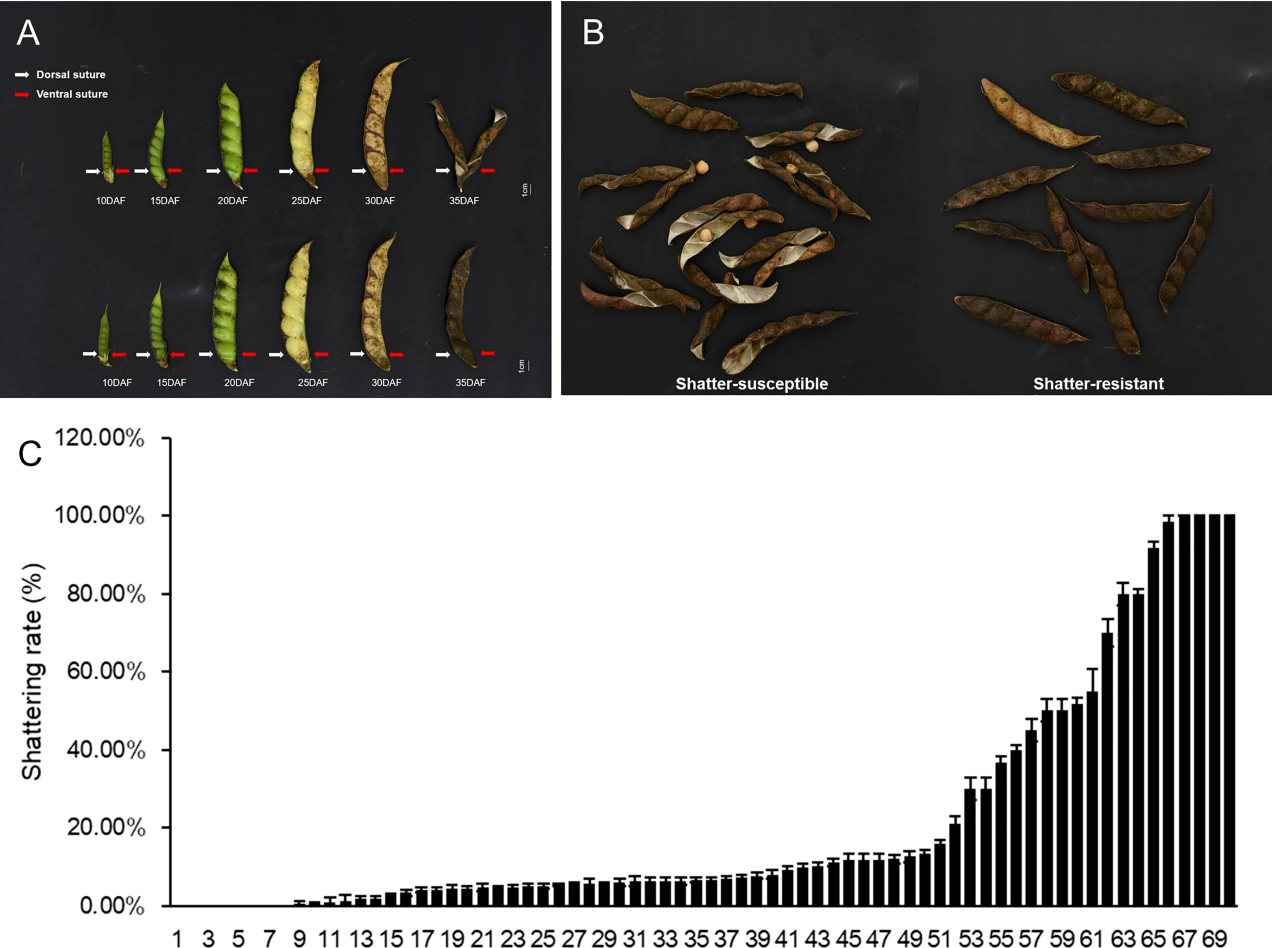
Different seed shattering habits of the pigeon pea accessions. **(A)** Different pods development stages of shatter-susceptible and shatter-resistant pigeon peas at 5, 10, 15, 20, 25, 30, and 35 days after flowering (DAF). **(B)** Schematic diagram of the types of shatter-resistant and shatter-susceptible pods of pigeon pea. **(C)** Shattering rate of 70 pigeon pea accessions. *Bars* indicate the mean ± SD.

Pod dehiscence was observed in the field (**Figures 2A, B**), and the highest seed shattering rate was 100% in 70 pigeon pea accessions (**Figure 2C**). Prior studies indicate that the ratio of actual seed yield to potential seed production for pigeon peas is quite low. We hypothesize that this phenomenon may be responsible for poor seed yield.

**Figure 3:**
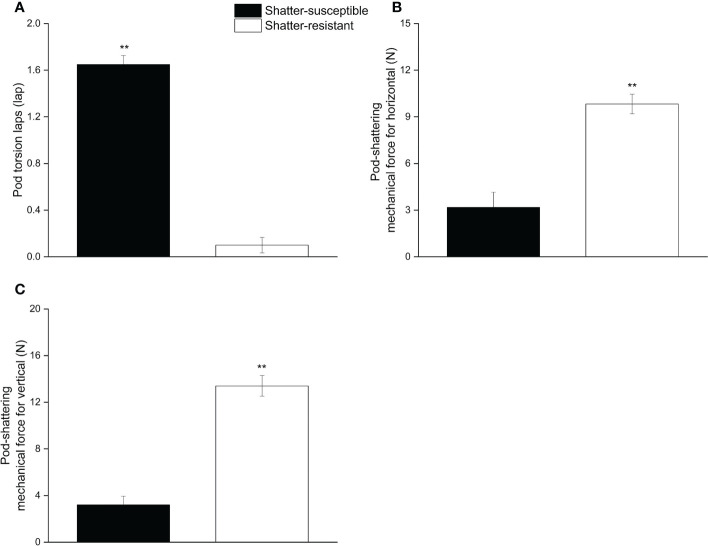
Pod mechanical characteristics of shatter-susceptible and shatter-resistant pigeon pea. **(A)** Pod wall torsion laps number. **(B)** Pod-shattering mechanical force for horizontal. **(C)** Pod-shattering mechanical force for vertical. ** Significant difference (*p*< 0.01) between shatter-susceptible and shatter-resistant pigeon peas.

The pod wall torsion lapse number, the horizontal and vertical pod mechanical forces were significantly different (*p<* 0.01) between shatter-resistant and shatter-susceptible pigeon pea accessions. The results showed that the pod wall torsion lapse number of shatter-susceptible pigeon pea accessions (1.65) was significantly (*p<* 0.01) higher than that of shatter-resistant pigeon pea accessions (0.01) (**Figure 3A**). Horizontal and vertical pod-shattering mechanical forces for shatter-resistant pigeon pea accessions (9.83 N, 13.40 N) were significantly (*p<* 0.01) higher than those of shatter-susceptible pigeon pea accessions (3.18 N, 3.21 N); mechanical forces were 3.09 and 4.17 times those of the shatter-susceptible pigeon pea accessions, respectively (**Figures 3B, C**).

### Morphological characteristics of pods

**Figure 4:**
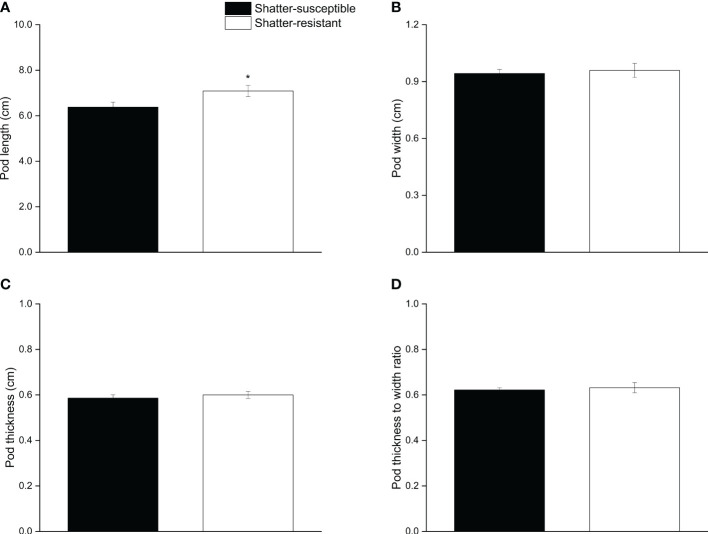
Pod morphological characteristics of shatter-susceptible and shatter-resistant pigeon pea. **(A)** Pod length. **(B)** Pod width. **(C)** Pod thickness. **(D)** thickness to width ratio. *Significant difference (p < 0.05) between shatter-susceptible and shatter-resistant pigeon peas.

The results for pod morphological characteristics showed that the pod length of shatter-susceptible pigeon pea accessions (6.37 cm) was significantly (*p<* 0.05) lower than that of shatter-resistant pigeon pea accessions (7.08 cm) (**Figure 4A**). Pod width, thickness, and the thickness-width ratio of shatter-resistant pigeon pea accessions were higher than those of shatter-susceptible pigeon pea accessions, but there were no significant differences (**Figures 4B–D**).

### Correlations between agronomic traits and pod-shattering rate

**Figure 5:**
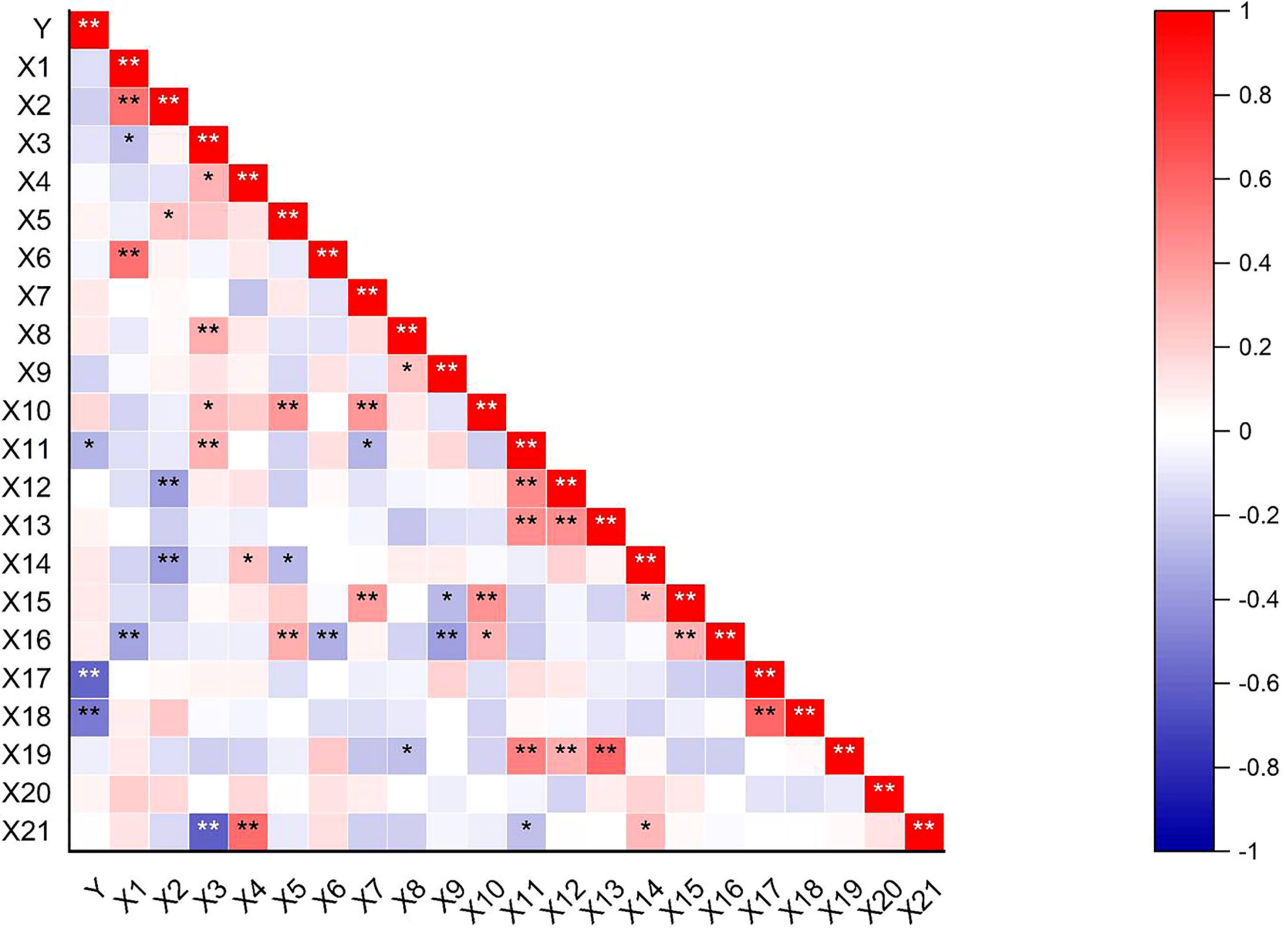
Heat map of correlation between agronomic traits and pod-shattering rate. Y = shattering rate; X1 = inflorescences per tiller; X2 = pods number per branch; X3 = ovules per flower; X4 = seeds per pod; X5 = seed weight per plant; X6 = plant height; X7 = crown breadth; X8 = length of the center of shape; X9 = width of the center of shape; X10 = phytomass; X11 = pod length; X12 = pod width; X13 = pod thickness; X14 = primary branches number; X15 = secondary branches number; X16 = fertile tillers number of per plant; X17 = pod-shattering mechanical force for horizontal; X18 = pod-shattering mechanical force for vertical; X19 = thousand-seed weight; X20 = flowers per inflorescence; X21 = setting rate. *Significant difference (p < 0.05) of probability, **Significant difference (p < 0.01) of probability.

Correlations between the shattering rate and 21 field agronomic traits showed that the pod shattering rate of pigeon pea was extremely significantly negatively correlated (*p<* 0.01) with the horizontal and vertical pod-shattering mechanical force. The pod shattering rate was significantly positively correlated (*p<* 0.05) with pod length (**Figure 5**).

### Histological analysis of pod ventral sutures

**Figure 6:**
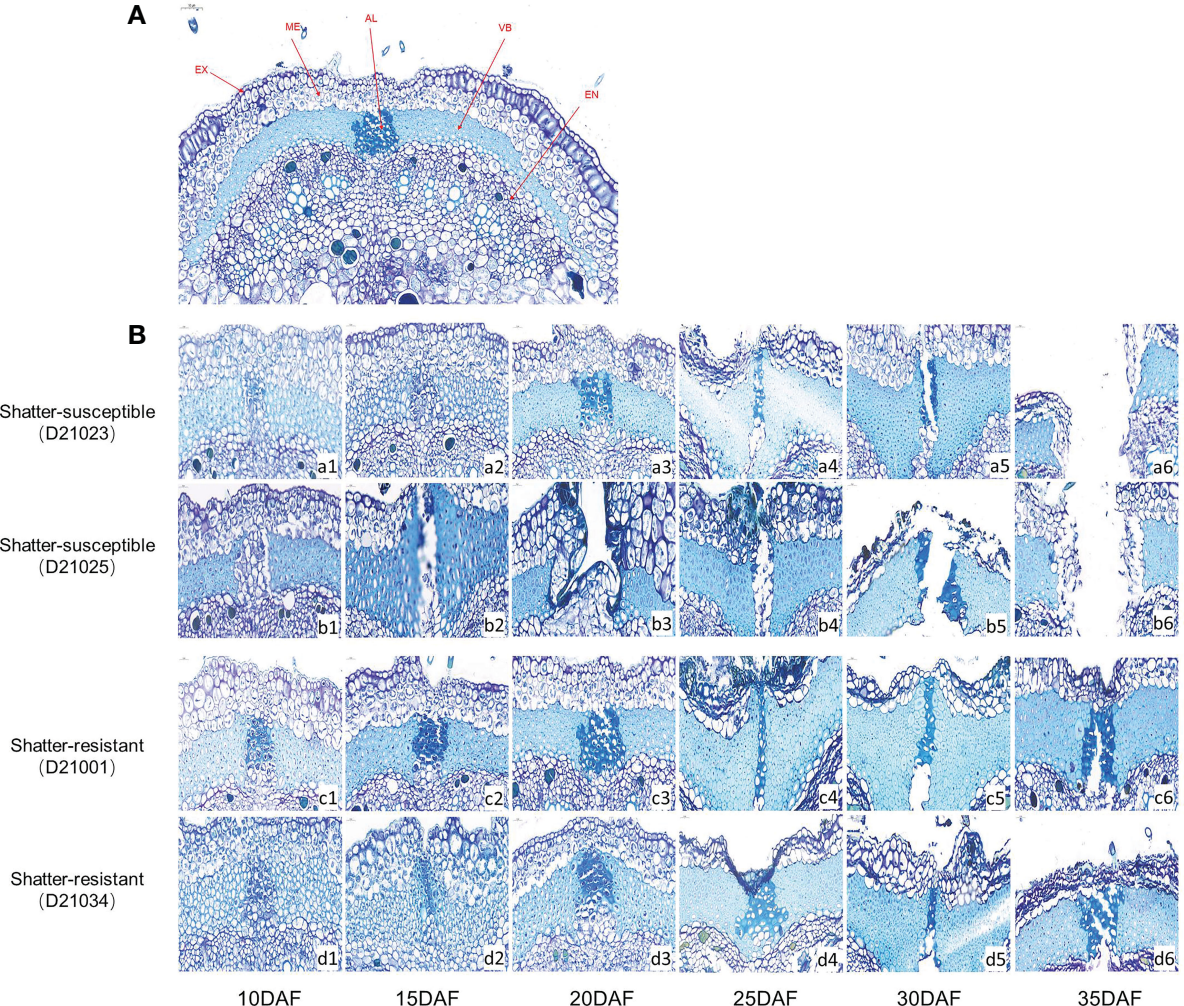
Histological analysis of the abscission zone. **(A)** Schematic diagram of the cross-section of the suture in the belly of pigeon pea pods. EX, Exocarp; ME, Mesocarp; EN, Exocarp; VB, Vascular bundle; AL, Abscission layer. **(B)** Cross-sectional structure of pod ventral sutures at different development stages of hatter-susceptible and shatter-resistant pigeon peas.

We compared the microstructure of ventral sutures (**Figure 6A**) in pigeon pea accessions at six developmental stages. The abscission layer cells of shatter-susceptible accessions initially formed at 10 DAF (**Figure 6B**, a1 and b1) and began to degrade at 15 DAF (**Figure 6B**, b2) outward from the mesocarp to the exocarp and inward to the endocarp (**Figure 6B**, a4 and b4). The pods had completely dehisced at 30 DAF (**Figure 6B**, a5 and b5). The shatter-resistant pod accessions also had an abscission layer that formed at 10 DAF (**Figure 6B**, c1 and d1). With the development of pods, the abscission layer degraded by 20 DAF (**Figure 6B**, c3 and d3) but without breaking through the endocarp and exocarp, and dehiscence was achieved at 35 DAF (**Figure 6B**, c6 and d6).

### Characterization of pod abscission layer cells

**Figure 7:**
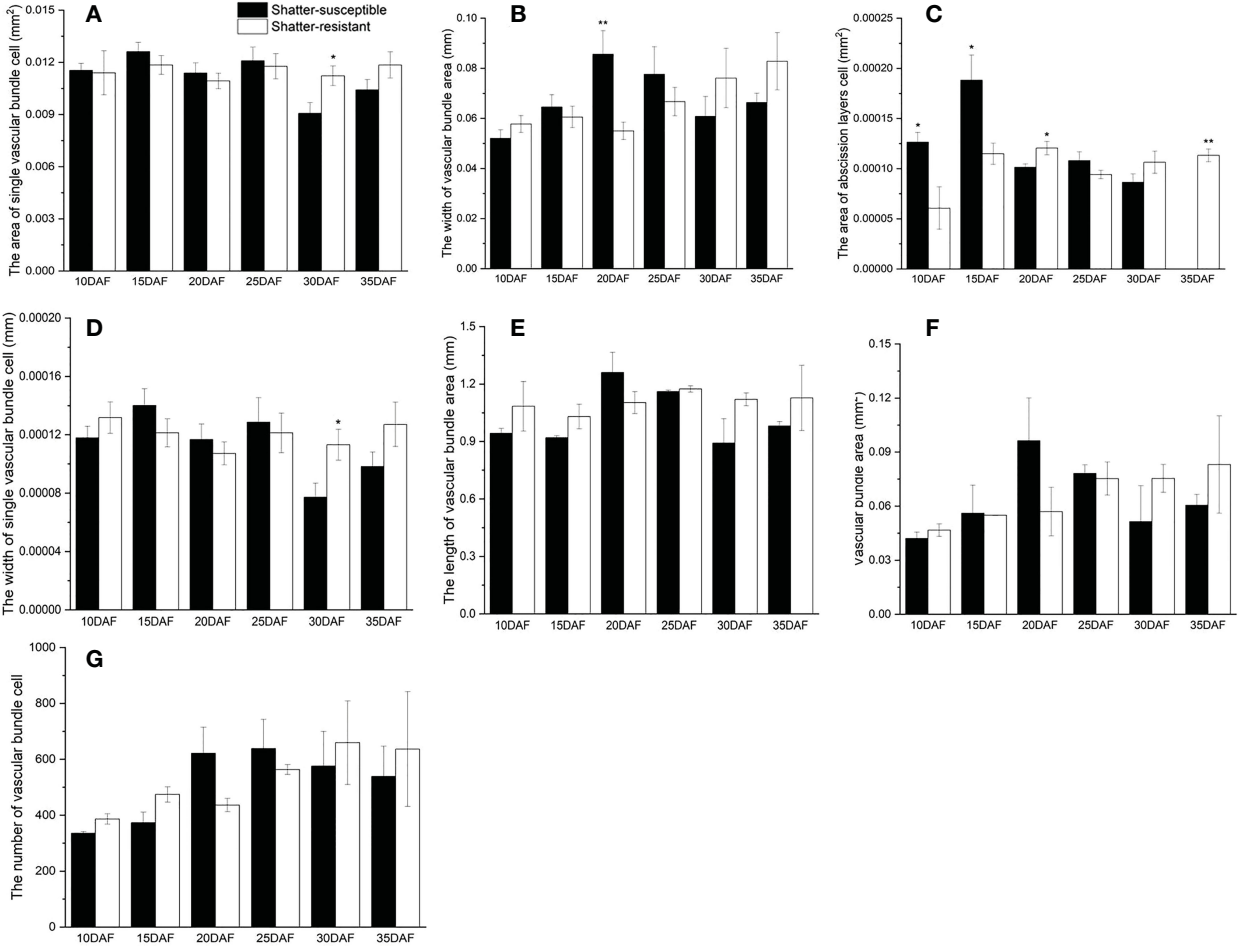
Characterization of pod abscission layer cells. **(A)** Area of single vascular bundle cell. **(B)** Width of vascular bundle area. **(C)** Area of abscission layers cell. **(D)** Width of single vascular bundle cell. **(E)** Length of vascular bundle area. **(F)** Vascular bundle area. **(G)** Number of vascular bundle cell. *Significant difference (p < 0.05), **Significant difference (p < 0.01) between shatter-susceptible and shatter-resistant pigeon peas.

The pod abscission layer cell characterization showed that the area of abscission layers cells and single vascular bundle cells, the width of single vascular bundle cells and the vascular bundle area were higher in shatter-resistant accessions than in shatter-susceptible accessions at 30 DAF (**Figure 7**). Single vascular bundle cell width and single vascular bundle cell area of shatter-resistant accessions were significantly (*p<* 0.05) higher than those of shatter-susceptible accessions at 30 DAF (**Figures 7A, D**). The width of the vascular bundle area of shatter-susceptible accessions was extremely significantly (*p<* 0.01) higher than that of shatter-resistant accessions at 20 DAF (**Figure 7B**). The abscission layer cell area of shatter-resistant accessions was extremely significantly (*p<* 0.01) higher than that of shatter-susceptible accessions at 35 DAF (**Figure 7C**).

In addition, the vascular bundle area, vascular bundle cell number and length of the vascular bundle area of shatter-susceptible accessions were lower than those of shatter-resistant accessions at 30 DAF and 35 DAF (**Figures 7E–G**).

**Figure 8:**
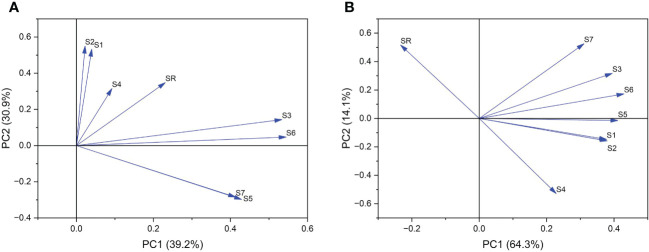
Principal component analysis of **(A)** pod growth stage (10, 15, 20 DAF) and **(B)** pod development stage (25, 30, 35 DAF). SR=shattering rate; S1= single vascular bundle cell width; S2=single vascular bundle cell area; S3= width of vascular bundle area; S4=abscission layers cell area; S5=the length of vascular bundle area; S6=vascular bundle area; S7=the number of vascular bundle cell.

Further principal component analysis was performed on 7 indices and the pod shattering rate. Six stages of pod development were divided into pod growth stage (10, 15, 20 DAF) (**Figure 8A**) and development stage (25, 30, 35 DAF) (**Figure 8B**). According to PCA of the pod growth stage, PC1 and PC2 explained 70.1% of the total variation in shatter-resistant and shatter-susceptible pigeon pea accessions. Single vascular bundle cell area and vascular bundle area were the most significant factors (**Figure 8A**). According to PCA of the pod development stage, PC1 and PC2 explained 75.4% of the total variation, and vascular bundle area and abscission layer cell area were the most significant factors affecting seed shattering (**Figure 8B**).

### Pod moisture variation

**Figure 9:**
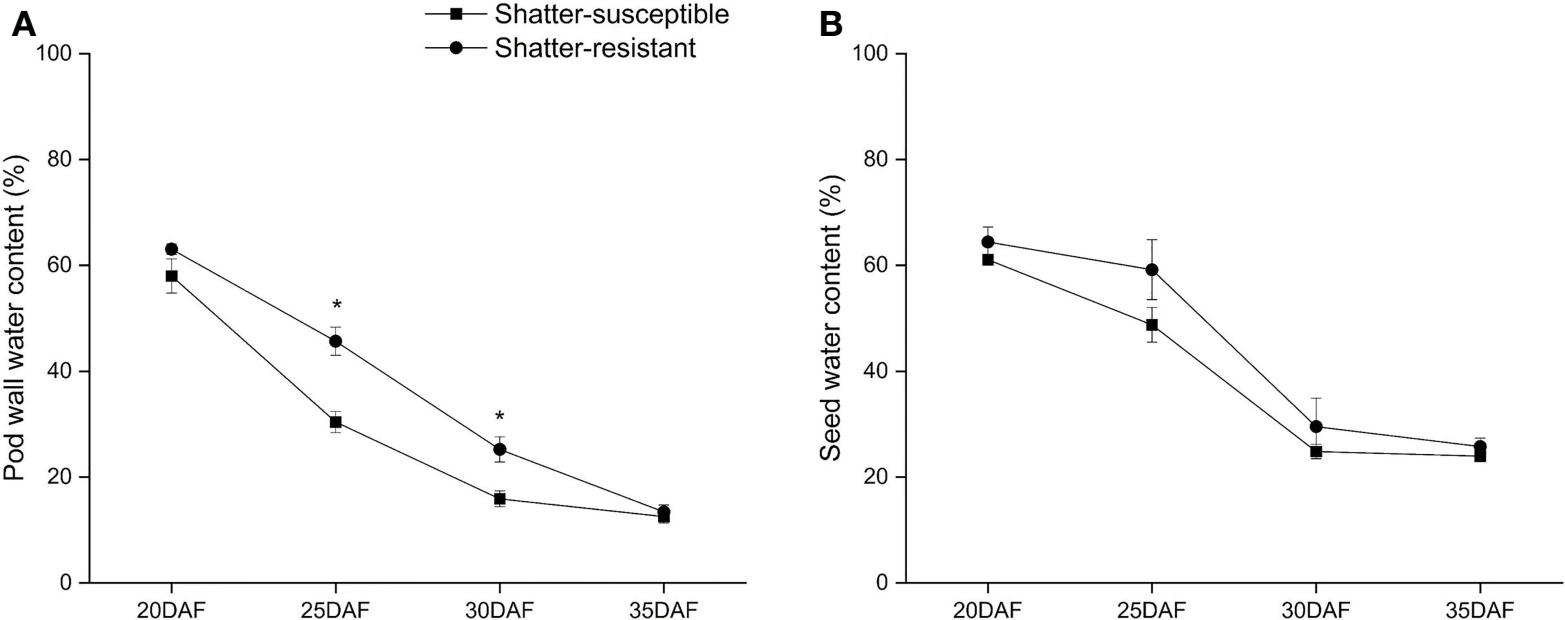
Pod wall water content **(A)** and seed water content **(B)** of shatter-susceptible and shatter-resistant pigeon peas accessions. *Significant difference (p < 0.05) between shatter-susceptible and shatter-resistant pigeon peas.

Comparing pod moisture variation for shatter-resistant and shatter-susceptible pigeon pea accessions at different development stages showed that the pod wall water content of shatter-susceptible accessions was lower than that of shatter-resistant accessions during pod development and reached significant difference (*p<* 0.05) at 25 DAF and 30 DAF (**Figure 9A**). The seed water content of shatter-resistant pigeon pea accessions was consistent with the trend of pod wall water content (**Figure 9B**).

### Hydrolytic enzyme activity

**Figure 10:**
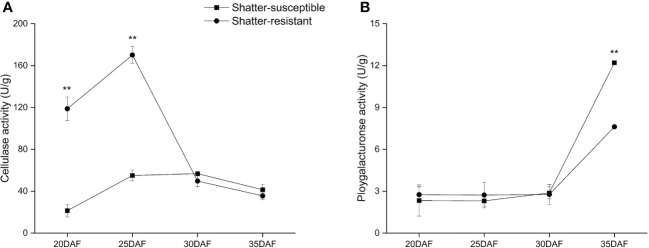
Specific activity of two cell wall-degrading enzymes, cellulase **(A)** and polygalacturonase **(B)** in the abscission zone. *Bars* indicate the mean ± SD. *Double asterisk* indicate a significant difference in the enzyme activity between shatter-susceptible and shatter-resistant accessions at the *p*< 0.01 level.

The CE activity showed the trend of first increasing and then decreasing. The CE activity of shatter-resistant accessions was significantly (*p<* 0.01) higher than that of shatter-susceptible accessions at 20 DAF and 25 DAF. However, the CE activity of shatter-resistant accessions decreased immediately after 25 DAF and was lower than that of shatter-susceptible accessions at 30 DAF and 35 DAF (**Figure 10A**).

The PG activity showed an increasing trend during pod development. The PG activity of shatter-susceptible accessions was lower than that of shatter-resistant accessions from 20 DAF to 30 DAF, but there was no significant difference. However, the PG activity of shatter-susceptible accessions increased immediately after 25 DAF and was significantly (*p<* 0.01) higher than that of shatter-resistant accessions at 35 DAF (**Figure 10B**).

## Discussion

### Yield components and seed shattering contribute to seed yield

Seed yield is controlled by yield components, other agronomic traits and environmental factors. In this study, the average ratio of actual seed yield to potential seed yield in pigeon pea was 6%, which was much lower than that of *Lolium perenne* (13%), *Dactylis glomerata* (17%), *Festuca arundinacea* (11%) or *Cleistogenes songorica* (43%) (Falcinelli, 1999; Li et al., 2014). Meanwhile, the highest seed shattering rate was 100% in 70 pigeon pea accessions. These results demonstrate that there is great potential to increase seed yield in pigeon pea, and higher seed shattering is one of the most important factors. Fertile tiller number per plant had a direct effect on the seed yield of pigeon pea, while the negative indirect effect of inflorescence number per tiller, based on the fertile tiller number per plant, was large. No indirect or direct effect was observed for flower number per inflorescence or thousand seed weight. These results differ from previous studies and show that seed yield in pigeon pea is not limited by the number of inflorescences, such as in cowpea (*Vigna unguiculata* (L.) Walp.) or red clover (*Trifolium pratense* L.) (Amdahl et al., 2017; Mashood et al., 2022). However, these results are similar to several other reports, which showed that fertile tiller number was the most important seed yield component in lentil (*Lens culinaris* Medik.) (Sharma et al., 2022) and chickpea (*Cicer arientinum* L.) (Ton and Anlarsal, 2017). These results indicate that decreasing seed shattering and increasing fertile tiller number will improve the actual seed yield of pigeon pea.

### Agronomic characters affect pod dehiscence

A previous study pointed out that in soybean, there was a strong correlation between the seed number per pod and pod dehiscence (Kataliko et al., 2019). In this study, we found that the horizontal and vertical pod-shattering mechanical forces, pod length, and number of tertiary branches were the main factors determining the pod-shattering rate of pigeon pea. By observing pod dehiscence characteristics, we found that shatter-resistant pigeon pea accessions were more capable of withstanding mechanical forces and lower pod torsion laps than shatter-susceptible pigeon pea accessions which was consistent with soybean (Funatsuki et al., 2014). Therefore, we inferred that the torsion force was the driving force, and pod torsion lapse was an intuitive performance feature of determining pod dehiscence in pigeon pea, which was consistent with a previous study (Dong et al., 2017b).

### Differences in histological and cytological characteristics in the abscission zone

Some researchers have noted that pod dehiscence is related to the cell tissue structure of pods (Tu et al., 2019; Kucek et al., 2020). To explore the development of the abscission zone in pigeon pea, we synthetic analyzed histological and cytological characteristics. There were obvious abscission layers in both shatter-susceptible and shatter-resistant pigeon pea accessions. Even during pod development, we found that shatter-susceptible and shatter-resistant pigeon peas had abscission layers at the same time. In addition, we also found that during the late development of pods, the vascular bundle area and vascular bundle cell number were the most significant negative factors affecting seed shattering. Therefore, it was speculated that larger vascular bundle tissue and cells were better able to withstand the dehiscence pressure from the abscission layer (Jia et al., 2021).

### Water content and hydrolytic enzyme activity

A previous study showed a correlation between pod water content and pod dehiscence (Menendez et al., 2019). The water content of pods can indirectly explain the ability of the vascular bundles of pods to transport nutrients (Zhang et al., 2018). The water content of the pod wall of shatter-resistant pigeon pea was significantly higher than that of shatter-susceptible pigeon pea at 25 DAF and 30 DAF in this study, indicating that at these times, the cell wall of shatter-susceptible pigeon pea had a higher degree of lignification. This result was also confirmed by histological staining.

CE and PG participate in the degradation of cellulose and pectin; they are two important hydrolase enzymes in the cell wall, and many studies have shown that these two enzymes can destroy the structure of plant cell walls and are closely related to pod dehiscence (Zhao et al., 2017; Dong et al., 2017a; Muriira et al., 2015). Previous studies have shown that the activities of these two enzymes reach a maximum at the pod maturity stage, and shatter-susceptible pod materials will transition from nondehiscence to dehiscence, while the opposite trend occurs in shatter-resistant pod materials (Christiansen et al., 2002).

In this study, the pods reached physiological maturity at 30 DAF. The CE and PG levels of shatter-susceptible pigeon pea began to be higher than those of shatter-resistant pigeon pea at this time, indicating that the two enzymes began to cleave the cell mass in shatter-susceptible pigeon pea, which destroyed the cell wall and caused the pods to dehisce.

### Model diagram

**Figure 11:**
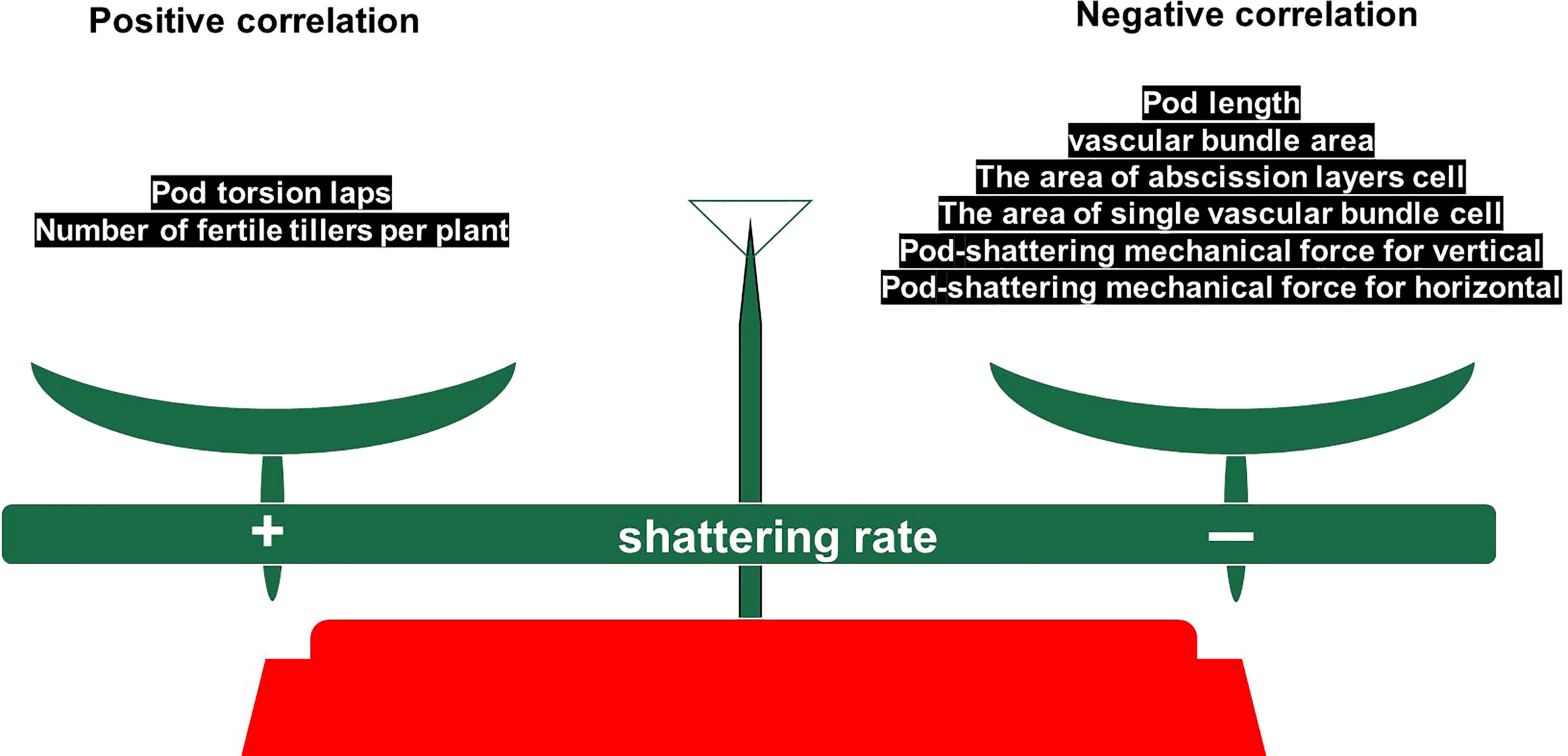
Balance conservation model.

We found that shatter-susceptible and shatter-resistant pigeon peas had abscission layers at the same time, but that abscission layer cells dissolved earlier in shatter-susceptible pigeon pea, which led to the tearing of the abscission layer. There was a positive correlation between pod torsion laps, tertiary branch number and dehiscence of pigeon peas. There was a negative correlation between pod length, single vascular bundle cell width, vertical and horizontal pod-shattering mechanical force, the single vascular bundle cell area and abscission layer cells, vascular bundle area and dehiscence of pigeon pea. In addition, we inferred that larger vascular bundle tissues and cells in the ventral suture of seed pods could effectively resist the dehiscence pressure of the abscission layer. Based on this, we propose a “balance conservation model” (**Figure 11**).

## Conclusion

In the present study, we found that there is great potential to increase seed yield in pigeon pea, and higher seed shattering is one of the most important factors. Fertile tiller number was the key component of seed yield. Multiplex morphology, histology, and cytological and hydrolytic enzyme activity analysis showed that shatter-susceptible and shatter-resistant pigeon peas had an abscission layer at the same time, but that abscission layer cells dissolved earlier in shatter-susceptible pigeon pea, which led to the tearing of the abscission layer. The vascular bundle cell number and vascular bundle area were the most significant negative factors affecting seed shattering. CE and PG were involved in the dehiscence process. In addition, we inferred that larger vascular bundle tissues and cells in the ventral suture of seed pods could effectively resist the dehiscence pressure of the abscission layer. This study provides a foundation for further molecular studies to increase the seed yield of pigeon pea.

## Data availability statement

The original contributions presented in the study are included in the article/supplementary material. Further inquiries can be directed to the corresponding authors.

## Author contributions

JZ and XD contributed to the conception and design of the study. XL, WS and QD performed the experiments. RH, RD and GL performed the statistical analysis. XL and WS wrote the first draft of the manuscript. All authors contributed to the article and approved the submitted version.
